# A Challenging Case of Severe Sickle Cell Crisis With Multiorgan Involvement: A Case Report

**DOI:** 10.7759/cureus.42437

**Published:** 2023-07-25

**Authors:** Rana Ibrahim, Abbas Fadel, Nour Sawli, Ali Mecheik

**Affiliations:** 1 Research Department, Saint George Hospital-Hadath, Beirut, LBN; 2 Infectious Diseases Department, Saint George Hospital-Hadath, Beirut, LBN; 3 Pharmacy Department, Saint George Hospital-Hadath, Beirut, LBN; 4 Intensive Care Unit Department, Saint George Hospital-Hadath, Beirut, LBN

**Keywords:** management challenges, blood exchange, acute chest syndrome, thrombotic events, multi-organ failure, septic shock, sickle cell anemia

## Abstract

Sickle cell anemia is a chronic and debilitating hemoglobinopathy characterized by the presence of abnormal hemoglobin, resulting in the formation of sickle-shaped red blood cells. This case report presents an unusual case of a 32-year-old female patient with sickle cell anemia who had not experienced any previous sickle cell crises since her diagnosis at the age of four years. Despite a stable clinical history, the patient's condition rapidly deteriorated, leading to septic shock, multiorgan failure, and atypical complications such as neurological impairment and acute kidney injury. Intensive management strategies, including blood exchange, mechanical ventilation, and aggressive antibiotic therapy, were implemented but unfortunately failed to reverse the progressive clinical deterioration. This case underscores the importance of early recognition and a multidisciplinary approach in managing atypical sickle cell crises to optimize patient outcomes. Further research is needed to improve our understanding and management of such presentations.

## Introduction

Sickle cell anemia is a hereditary hemoglobinopathy characterized by the presence of abnormal hemoglobin, specifically hemoglobin S, which leads to the formation of sickle-shaped red blood cells (RBCs). It is a chronic and debilitating condition associated with recurrent vaso-occlusive crises, hemolytic anemia, and end-organ damage [1]. Although sickle cell crises are common in patients with sickle cell anemia, their presentation and severity can vary significantly, ranging from mild pain episodes to life-threatening complications [2]. The management of sickle cell anemia crises involves a multifaceted approach, including pain management, supportive care, blood transfusions, and antibiotic therapy [3]. Additionally, complications such as acute chest syndrome and thrombotic events may further complicate the clinical course and require targeted interventions [4,5]. However, despite advancements in the understanding and management of sickle cell anemia, certain cases can present with atypical features and unpredictable outcomes, posing significant clinical challenges [6]. Furthermore, we highlight the importance of a multidisciplinary approach involving hematologists, intensivists, infectious disease specialists, and other healthcare professionals to optimize the management and outcomes of patients with sickle cell anemia [7,8]. By sharing this case, we hope to contribute to the existing knowledge base and stimulate further investigations to enhance the care of patients with sickle cell anemia. We present the case of a 32-year-old female patient with a known history of sickle cell anemia who presented to the emergency department with non-specific pain and fever. Despite a relatively stable clinical course with no previous sickle cell crises since the initial diagnosis of sickle cell anemia, the patient's condition rapidly deteriorated, ultimately leading to septic shock and multiorgan failure.

## Case presentation

**Figure 1:**
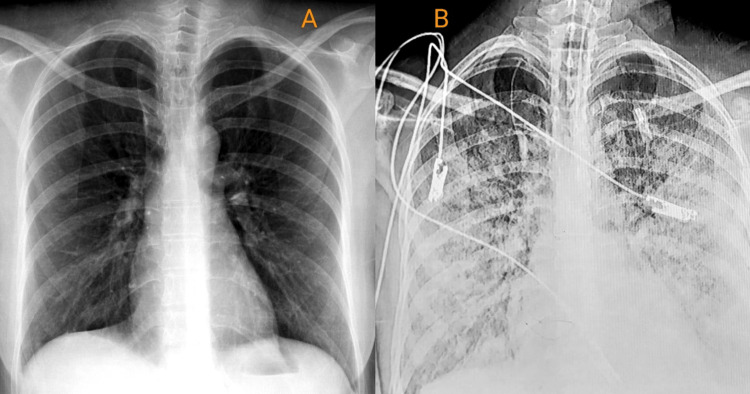
Evolution of mild bilateral lung scatter in chest x-rays: day one (A) versus day 10 (B).

A 32-year-old female patient presented to the emergency department of Saint George's Hospital with symptoms of generalized fatigue, muscle pain, headache, fever, sore throat, and runny nose. The patient had a previous diagnosis of sickle cell anemia at the age of four, although she had not experienced any sickle cell crises in the past. She had no history of smoking, alcohol consumption, or home medications but had undergone three C-section deliveries. Upon admission, the patient's vital signs were stable, including a normal temperature, heart rate, respiratory rate, blood pressure, and oxygen saturation. Upon physical examination, the patient exhibited an erythematous throat but lacked palpable lymphadenopathy or any skin manifestations. The chest and abdomen examinations yielded normal results, and the neurologic exam showed no abnormalities, including no signs of neck stiffness. Initial laboratory investigations indicated microcytic anemia (white blood cells (WBC)=5.04 k/μL, neutrophils (NEU)=52.5%, lymphocytes (LYM)=39.1%, monocytes (MONO)=5.4%, eosinophils (EOS)=2.2%, basophils (BASO)=0.8%, RBC=3.61 M/μL, hemoglobin=8.3 g/dL, hematocrit=23.1%, mean corpuscular volume (MCV)=64.0 fL, mean corpuscular hemoglobin (MCH)=23.0 pg, mean corpuscular hemoglobin concentration (MCHC)=35.9 g/dL, red blood cell distribution width (RDW)=14.6%, and platelets=162 k/μL), consistent with her history of sickle cell anemia, suggesting a possible sickle cell crisis. To confirm the diagnosis and estimate the severity of sickling, hemoglobin electrophoresis and sickling slide tests were requested. The results revealed a high level of hemoglobin S (HbS) at 70%, indicating a severe sickle cell crisis. Empirical treatment with ceftriaxone (two grams every 12 hours) was initiated due to the presence of a fever and a congested throat. Additionally, nasal swab cultures and polymerase chain reaction (PCR) tests for COVID-19 and influenza were obtained to rule out viral infections. The patient received vigorous hydration and a blood transfusion as per the sickle cell crisis management protocol. Despite these interventions, her fever persisted, and she developed hypoxia, requiring supplemental oxygen via a nasal cannula at two liters per minute. The following day, the patient experienced severe hip pain, increased fatigue, and a headache, prompting further investigations. A CT scan of the brain was performed to rule out bleeding or strokes, and the results were negative for these conditions. A chest x-ray revealed the presence of mild bilateral lung scatter, which has exhibited progressive deterioration within days (Figure 1).

**Figure 2:**
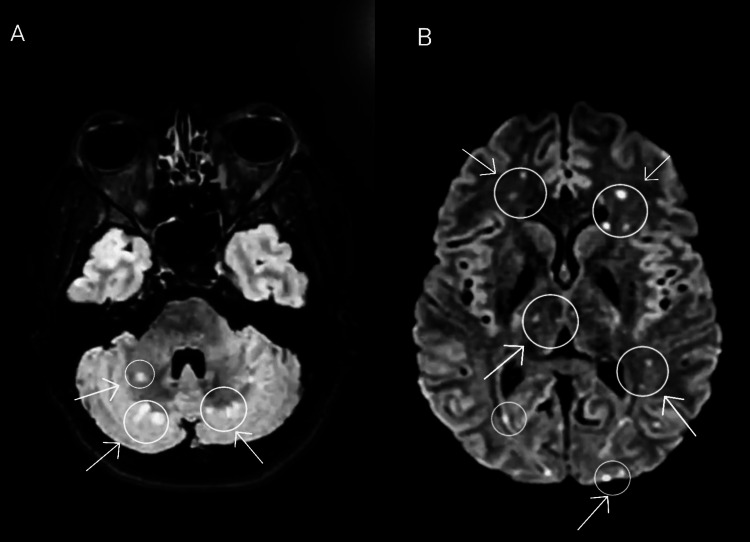
Multiple infarcts were observed in supra and infratentorial areas on brain MRI.

Due to the deterioration of her neurological condition, as evidenced by a Glasgow Coma Scale (GCS) score of five out of 15, indicating severe neurological impairment, the decision was made to transfer the patient to the intensive care unit (ICU) for enhanced monitoring and specialized medical attention. Subsequently, an antiphospholipid test was conducted, yielding a positive outcome. To exclude the possibility of meningitis, a lumbar puncture and cerebrospinal fluid analysis were performed. The brain MRI results revealed the presence of multiple infarcts in the supra and infratentorial areas, as depicted in Figure 2.

Furthermore, the patient experienced another episode of acute chest syndrome. Despite thorough investigations, the underlying cause of the persistent fever remained unidentified, prompting consideration of several potential etiologies, including septic and central causes. Given the gravity of the clinical presentation and the suspicion of a severe sickle cell crisis, the decision was made to intubate the patient and perform a blood exchange with erythropheresis. Before the procedure, there were three units of blood transfused, then 14 units during the exchange, and then eight more units afterward. In the intensive care unit (ICU), the patient's antibiotic regimen was intensified, initially with two grams of meropenem every eight hours. Still, due to the persistence of the fever, empirical replacement therapy with ceftazidime/avibactam every eight hours was initiated. Additionally, echocardiography was performed due to tachycardia, revealing an ejection fraction of 60%.

In relation to the patient's ventilation status, a computed tomography pulmonary angiogram (CTPA) was performed, and the results were negative. The individual presented with progressive manifestations of acute respiratory distress syndrome (ARDS), coupled with an escalated oxygen requirement on the mechanical ventilator and marked clinical deterioration. Concurrently, sequential chest radiographs demonstrated a worsening pattern, as previously described, ultimately resulting in challenging ventilation management. To address this issue, the patient received comprehensive sedation and intermittent neuromuscular blockade as necessary. It is important to note that the patient has a recessive trait of thalassemia but has not received specific therapy for sickle cell anemia. Following a ten-day intensive care unit (ICU) admission, the patient manifested hypotension along with a persistent fever. Simultaneously, laboratory investigations revealed a progressive development of acute kidney injury, as evidenced by an increase in serum creatinine levels to 2.6 mg/dL. Given the clinical presentation, the medical team suspected septic shock and promptly obtained new cultures. The patient developed acute kidney failure, as evidenced by an elevated creatinine level and hyperphosphatemia, hyperkalemia, and hypocalcemia. Cultures obtained revealed clearance of infections, except for the presence of extended-spectrum beta-lactamase (ESBL) in the rectum. Considering the patient's deteriorating clinical status, the medical team suspected uncontrolled vaso-occlusive disease leading to organ damage. Despite diligent attempts to manage the patient's condition, encompassing the administration of suitable antibiotics and comprehensive supportive measures such as high-dose vasopressors and stress-dose corticosteroids with judicious fluid management, the patient's renal failure remained uncontrolled. Regrettably, the patient succumbed to profound septic shock, ultimately resulting in her demise.

## Discussion

This study adds to the growing body of evidence on atypical presentations of sickle cell anemia crises, highlighting the need for improved recognition and management of such cases. While previous studies have focused on classical manifestations, there is a lack of literature addressing atypical presentations and their unique features, including the absence of acute pain and the development of complications such as thrombotic events and acute chest syndrome [9,10]. Understanding the heterogeneous nature of sickle cell disease is crucial, as it presents a wide range of clinical manifestations [11,12]. Diagnostic challenges are encountered in identifying atypical sickle cell crises, as evidenced by the delayed diagnosis in this patient. By elucidating the diagnostic workup, management strategies, and outcomes of this case, healthcare providers can enhance their knowledge and awareness, leading to earlier recognition and appropriate intervention in future cases [13,14]. Considering atypical presentations of sickle cell crises is essential, particularly in patients with sickle cell anemia who present with non-specific symptoms. A comprehensive diagnostic workup, including laboratory tests and imaging studies, is necessary to identify potential complications and underlying causes. A multidisciplinary approach involving various healthcare professionals optimizes management and improves patient outcomes [15,16]. While the effectiveness of blood exchange in severe sickle cell crises has been reported in previous studies, its application in cases with atypical presentations and complications remains understudied. This study documents the successful implementation of blood exchange in a patient with atypical sickle cell crises, providing valuable insights into management strategies [17,18].

Regarding the relationship between sickle cell anemia and antiphospholipid antibodies, the current body of scientific research is limited, and a consensus regarding the exact prevalence and clinical significance of this association has not yet been reached. Nevertheless, several studies have reported an increased prevalence of antiphospholipid antibodies in individuals with sickle cell disease compared to the general population. For instance, a study published in Thrombosis and Hemostasis in 2014 revealed that approximately 30% of children diagnosed with sickle cell anemia had positive antiphospholipid antibodies [19]. Similarly, a study published in the American Journal of Hematology in 2016 documented a similar prevalence of antiphospholipid antibodies among adults with sickle cell anemia [20]. The presence of antiphospholipid antibodies in individuals with sickle cell anemia has been associated with an elevated risk of developing specific complications, including vaso-occlusive crises, acute chest syndrome, and stroke [19,20]. However, the precise underlying mechanisms explaining this association remain incompletely understood. It is worth noting that these studies have provided important insights into the prevalence of antiphospholipid antibodies in individuals with sickle cell anemia and their potential impact on disease complications. However, further research is needed to better understand the mechanisms and clinical significance of this association. Studies with larger sample sizes and diverse populations would contribute to a more comprehensive understanding of the relationship between vaso-occlusive diseases and antiphospholipid antibodies.

This case highlights the importance of early recognition, aggressive management, and close monitoring in patients with sickle cell anemia, particularly during sickle cell crises. A multidisciplinary approach involving hematologists, intensivists, infectious disease specialists, and other healthcare professionals is crucial to optimizing patient outcomes. To prevent similar cases and optimize outcomes in individuals with sickle cell anemia, comprehensive patient education programs should be implemented, empowering patients with knowledge and skills for self-management and early crisis recognition. Individualized care plans tailored to each patient's needs should be developed, incorporating preventive measures and addressing psychosocial aspects of care. Consideration should be given to the use of hydroxyurea therapy, a proven intervention for reducing the frequency of acute sickle cell pain and acute chest syndrome, reducing the need for blood transfusions and hospitalizations and possibly improving survival. For informed decision-making and appropriate interventions, genetic counseling and prenatal screening should be offered to prospective parents. Comprehensive health maintenance, including regular monitoring of laboratory parameters and management of comorbidities, is paramount. Early detection and effective management of thrombotic events in sickle cell disease rely on recognizing alarming signs and closely monitoring HbS levels. Neurological symptoms indicating thrombotic involvement in the cerebral vasculature should be promptly addressed.

Maintaining HbS levels below 30% is crucial to reducing thrombotic complications in sickle cell disease, as advised by the American Society of Hematology (ASH). This can be achieved through regular medical monitoring, taking prescribed medications like hydroxyurea or L-glutamine, making healthy lifestyle choices, and seeking education and support. Consulting with a healthcare provider for personalized guidance is essential to managing the disease effectively and maintaining HbS levels within the recommended range. Adhering to these guidelines allows healthcare providers to minimize severe crises, improve outcomes, and enhance the quality of life for individuals with sickle cell anemia. Individualized assessment of anemia and HbS percentage is recommended for transfusion therapy decisions. Regular monitoring of hemoglobin levels is necessary to manage the hyperviscosity of the blood and minimize the risk of end-organ damage. For HbS levels above 30%, personalized treatment and consideration of blood apheresis are essential preventive measures against life-threatening vaso-occlusive disease. Early intervention is emphasized, even in cases with normal brain imaging, highlighting the importance of considering clinical symptoms and imaging findings.

Further research is imperative to ascertain the specific patient population that would derive substantial benefits from apheresis as a primary prevention intervention in sickle cell disease. It is crucial to conduct controlled clinical trials and large-scale studies employing rigorous methodologies to establish compelling evidence concerning essential aspects such as patient selection criteria, disease severity stratification, and treatment outcomes. These studies should encompass meticulous evaluations of factors including age, genotype, comorbidities, and medical history to ascertain their potential as predictors of treatment response. Comprehensive assessments of disease severity and long-term outcomes are warranted to determine the optimal timing, frequency, and duration of apheresis interventions. Systematic collection of clinical data, encompassing laboratory parameters, imaging findings, and patient-reported outcomes, would significantly contribute to a comprehensive understanding of the intervention's impact on disease progression and overall patient well-being. Ultimately, the synthesis of robust evidence derived from well-executed studies would facilitate the development of evidence-based guidelines, thereby providing precise recommendations for the judicious utilization of apheresis as a primary prevention strategy in the management of sickle cell disease.

## Conclusions

This case report sheds light on the atypical presentation of sickle cell crises and the challenges encountered in their diagnosis and management. By highlighting the diverse clinical manifestations, complications, and need for multidisciplinary care, this report emphasizes the importance of recognizing and addressing atypical sickle cell crises promptly. Further research is necessary to better understand the underlying mechanisms and develop tailored management strategies to improve outcomes in these complex cases.
